# Combined Fishing and Climate Forcing in the Southern Benguela Upwelling Ecosystem: An End-to-End Modelling Approach Reveals Dampened Effects

**DOI:** 10.1371/journal.pone.0094286

**Published:** 2014-04-07

**Authors:** Morgane Travers-Trolet, Yunne-Jai Shin, Lynne J. Shannon, Coleen L. Moloney, John G. Field

**Affiliations:** 1 IFREMER, Fisheries Laboratory, Boulogne sur Mer, France; 2 IRD CRH UMR EME 212, Sète, France; 3 Marine Research Institute, University of Cape Town, Rondebosch, South Africa; University of Vigo, Spain

## Abstract

The effects of climate and fishing on marine ecosystems have usually been studied separately, but their interactions make ecosystem dynamics difficult to understand and predict. Of particular interest to management, the potential synergism or antagonism between fishing pressure and climate forcing is analysed in this paper, using an end-to-end ecosystem model of the southern Benguela ecosystem, built from coupling hydrodynamic, biogeochemical and multispecies fish models (ROMS-N_2_P_2_Z_2_D_2_-OSMOSE). Scenarios of different intensities of upwelling-favourable wind stress combined with scenarios of fishing top-predator fish were tested. Analyses of isolated drivers show that the bottom-up effect of the climate forcing propagates up the food chain whereas the top-down effect of fishing cascades down to zooplankton in unfavourable environmental conditions but dampens before it reaches phytoplankton. When considering both climate and fishing drivers together, it appears that top-down control dominates the link between top-predator fish and forage fish, whereas interactions between the lower trophic levels are dominated by bottom-up control. The forage fish functional group appears to be a central component of this ecosystem, being the meeting point of two opposite trophic controls. The set of combined scenarios shows that fishing pressure and upwelling-favourable wind stress have mostly dampened effects on fish populations, compared to predictions from the separate effects of the stressors. Dampened effects result in biomass accumulation at the top predator fish level but a depletion of biomass at the forage fish level. This should draw our attention to the evolution of this functional group, which appears as both structurally important in the trophic functioning of the ecosystem, and very sensitive to climate and fishing pressures. In particular, diagnoses considering fishing pressure only might be more optimistic than those that consider combined effects of fishing and environmental variability.

## Introduction

Marine ecosystems are affected by multiple factors, natural and anthropogenic, interacting together and making ecosystem dynamics difficult to understand and predict. Climate change is now a well established phenomenon [1] and its observed effects on marine ecosystems range from shifts in species distribution [2], [3] or phenology [4] to extreme habitat perturbations such as coral reef bleaching [5]. On the other hand, fishing has been demonstrated to affect directly the abundance and demographic structure of target species, possibly leading to species collapse [6], [7], and to indirectly affect the entire ecosystem through predation and competitive interactions [8], [9], [10]. These indirect effects can disrupt the size structure of fish communities [11], the mean trophic level [12] or lead to the proliferation of undesirable species such as jellyfish [13].

Fishing pressure together with climate variability and change can affect the whole food web due to propagation of their direct effects through top-down and bottom-up controls [14], [15]. Despite the intensity and range of their individual effects, one of the growing concerns is the difficulty of predicting the ecosystem response to simultaneous changes in both climate and fishing drivers [16], [17], [18], as their interaction could lead to synergistic effects, i.e. be stronger than the isolated impact of each perturbation. With the worldwide objective of sustainable fisheries stated during the 2002 World Summit for Sustainable Development in Johannesburg, as well as the preservation of the good environmental status of the seas (as required by the European Marine Strategy Framework Directive), it is necessary to better understand how these factors may simultaneously affect marine ecosystems in order to manage marine activities in a more integrative way.

Field data have been used to disentangle fishing and climate effects using fish time series [19], [20], [21], with the underlying hypothesis that climate and exploitation effects are additive. More process-based studies have investigated how climate and fishing pressure interact and eventually affect ecosystems; Prince and Goodyear [22] show that a shallowing oxycline reduces the vertical habitat of tuna, making them more catchable by fisheries. Conversely, through its effect on intrinsic growth rates, fishing seems to magnify fluctuations in fish abundance [23]. In their review, Planque et al. [24] describe how the effects of fishing may induce changes in the ecosystem response to climate change or variability, due to reduced resilience, demographic changes, selection of particular sub-units of fish stock and/or increased turnover rates.

To study the potential synergistic or antagonistic effects induced by simultaneous changes in fishing pressure and climate, an alternative to analysis of field data time series is to use ecosystem models as virtual laboratories, where forcing variables can be controlled and information at different levels of the ecosystem can be tracked (e.g. [18]). In order to assess climate and fishing impacts on marine ecosystems, these processes must be considered explicitly in the model as well as potential feedbacks, as is the case with the recent development of end-to-end models [25], [26]. Here we use an end-to-end model coupling hydrodynamic, biogeochemical and multispecies fish models and apply it to the Benguela upwelling ecosystem, to provide understanding of how fishing and climate effects combine through the food web. Our study will complement previous modelling experiments in the Benguela system which have suggested that a heavily fished ecosystem may be more likely to be bottom-up controlled by the environment [27], [28], [29]. The end-to-end model used in this study has been applied previously to the southern Benguela ecosystem, and has been largely documented for this ecosystem [30], [31], [32], [33].

## Material and Methods

The end-to-end model used in this study consists of three component models, representing hydrodynamics, plankton dynamics and multiple fish species dynamics. These component models have been fully described in previous publications, so they are only summarized here with some additional details provided in the supporting information.

### The physical model ROMS

The physical environment of the Benguela ecosystem is represented through the Regional Ocean Modeling Systems (ROMS), using the configuration developed by Penven et al. [34]. Resolving the Navier-Stokes equation, it simulates the 3D currents of the southern Benguela upwelling and the Agulhas Bank on a curvilinear grid from 40°S to 28°S and from 10°E to 24°E, with 20 sigma vertical layers. Forcing variables are extracted from the COADS (Comprehensive Ocean-Atmosphere Data Set; [35]) monthly climatology and include winds, heat and salinity fluxes.

### Biogeochemical model of nutrients and plankton dynamics

**Figure 1:**
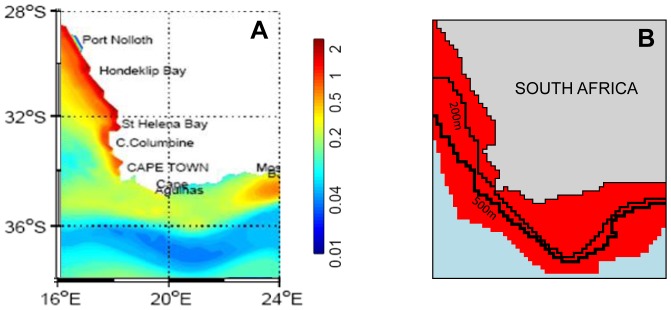
Spatial characteristics of the coupled models. (a) Annual primary production (in gC.m^−2^.d^−1^) in the upper 65 m simulated by ROMS-N_2_P_2_Z_2_D_2_ (adapted from [36]) and (b) spatial extent of fish individuals modeled in OSMOSE, aggregated over species, ages and seasons with delimitation of the 200 m and 500 m bathymetry.

ROMS has been coupled online to a N_2_P_2_Z_2_D_2_ biogeochemical model [36] which represents two compartments of nutrients (N), phytoplankton (P), zooplankton (Z) and detritus (D). Each plankton compartment can be assigned to a functional group which is characterized by a specific size range: flagellates (2–20 μm) and diatoms (20–200 μm) for phytoplankton, and ciliates (20–200 μm) and copepods (200–3000 μm) for micro-zooplankton and meso-zooplankton, respectively. This low trophic levels (LTL) model represents fluxes of nitrogen among compartments according to a number of modelled processes (see [36] and table S1 for model equations and parameters), some of which are elaborated below. Uptake of nutrients by phytoplankton groups assumes a preference for ammonium over nitrate. Copepods predate both phytoplankton groups, with a higher efficiency for diatoms; conversely smaller ciliates show a higher efficiency when grazing on flagellates. Unassimilated phytoplankton goes to the detritus compartments (egestion) and excretion is represented by nitrogen flux from zooplankton groups towards the ammonium pool. Remineralisation completes the link between detritus and nutrient compartments (the latter being linked through nitrification). Constant mortality terms are applied to phytoplankton and zooplankton groups. In addition to advection due to currents, the physical model impacts phytoplankton growth through light (derived from irradiance and phytoplankton concentration) and temperature. The coupled model ROMS-N_2_P_2_Z_2_D_2_ allows representation of the general features of the system, i.e. wind-driven upwelling on the West coast of South Africa characterized by high primary production and a relatively less productive area in the South, over the Agulhas Bank (figure 1a).

### Modelling the high trophic levels (HTL) with OSMOSE

**Figure 2:**
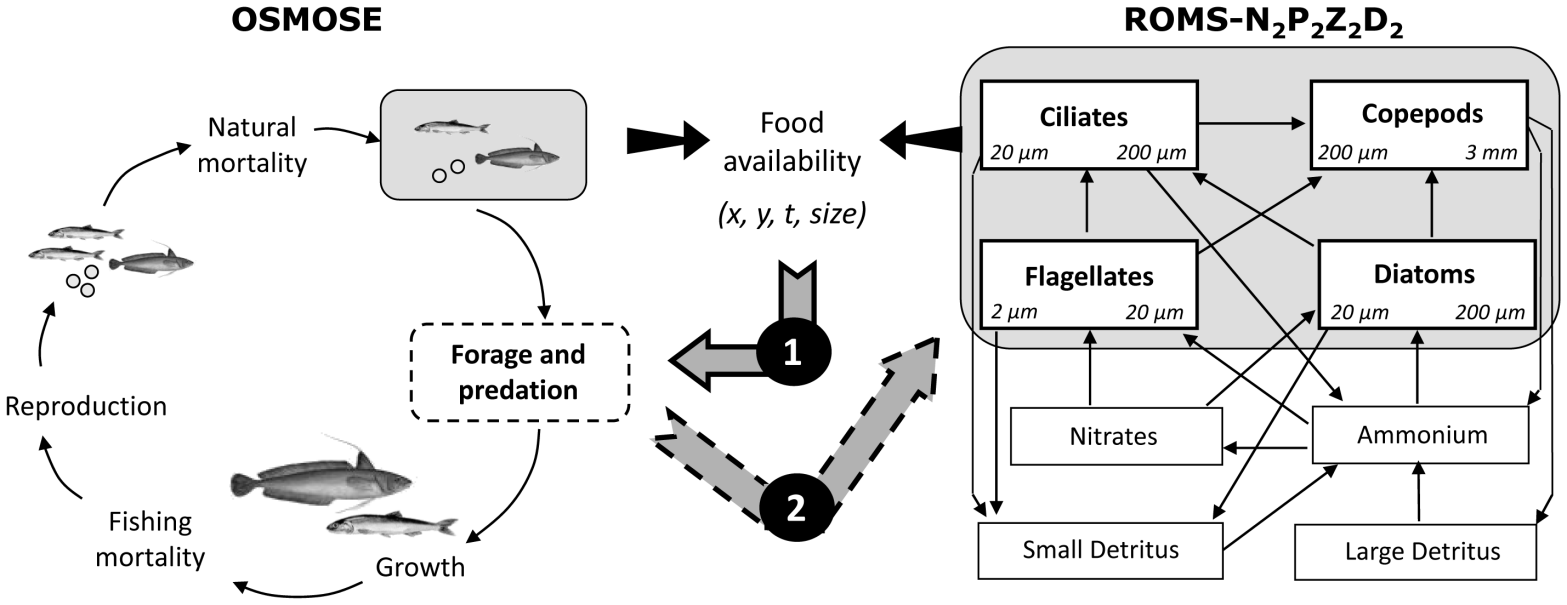
Processes represented within and for coupling the model components. Processes modelled within a time step (15 days) in OSMOSE (left hand side) and fluxes represented between functional groups in N_2_P_2_Z_2_D_2_ (right hand side). Coupling of models occurs through the predation process, where plankton biomass serves as a prey field for fish schools (arrow 1), and an explicit fish-induced predation mortality is applied as feedback on plankton groups (arrow 2).

OSMOSE (Object-oriented Simulator of Marine ecOSystems Exploitation, [37], [38]) is a multispecies model representing the whole life cycle of several species of fish, from eggs and larvae to juveniles and adults, which explicitly takes into account growth, predation, reproduction, natural and starvation mortalities as well as fishing mortality (figure 2, table S2). This individual-based model (IBM) simulates fish schools interacting in a two-dimensional grid and is based on opportunistic and size-based predation. The predation process occurs when there are both spatio-temporal co-occurrence and size compatibility between a predator and its prey. Thus the food web structure emerges from these local individual interactions [31]. Predation success, defined as prey biomass eaten over the maximum biomass a predator can feed upon, has repercussions on the school growth rate and mortality: if the maintenance requirements are not fulfilled by the amount of ingested food the number of fish constituting the school decreases exponentially with the starvation mortality rate. When the biomass of prey eaten is higher than maintenance requirements, the growth rate of fish is positive, following a product function of the von Bertalanffy growth rate varying with fish age, and the predation success. Predation success has also an indirect effect on the reproduction process through the biomass of spawners which, combined with relative fecundity parameters, will define the number of eggs released in the system. Fishing pressure is represented through a constant fishing mortality per species, affecting the number of fish per school when older than the specified age at recruitment.

OSMOSE has been applied to the Benguela ecosystem on a 2D regular grid (figure 1b) where it simulates the dynamics of one euphausiid species and ten fish species from small pelagic fish to large demersal fish [31], [30] (table S3): euphausiid (*Euphausia lucens*), lanternfish (*Lampanyctodes hectoris*), lightfish (*Maurolicus muelleri*), anchovy (*Engraulis encrasicolus*), sardine (*Sardinops sagax*), redeye (*Etrumeus whiteheadi*), horse mackerel (*Trachurus trachurus capensis*), deep water Cape hake (*Merluccius paradoxus*), shallow water Cape hake (*Merluccius capensis*), snoek (*Thyrsites atun*) and silver kob (*Argyrosomus inodorus*). Calibration was undertaken using a dedicated genetic algorithm [39], [32], [33]) where the unknown larval mortalities of fish species are estimated in order to fit fish biomasses observed in the 1990s [40]. The time step was set to 15 days, which allows representation of seasonal dynamics.

### Coupling ROMS-N_2_P_2_Z_2_D_2_ and OSMOSE

The HTL OSMOSE model has a 2-ways coupling to the ROMS-N_2_P_2_Z_2_D_2_ model described above through an opportunistic predation process [32], [31], [33]. Phytoplankton and zooplankton compartments (variable in space and time) are used as potential food for fish in addition to co-occurring ichthyoplankton which are modelled in OSMOSE. The biomass of each plankton group is considered homogeneous over their size range. Thus at each time step, predator fish schools feed upon both other co-occuring fish schools of suitable size leading to an explicit mortality of these schools and plankton groups if their size range allows it. As feedback, a fish-induced explicit predation mortality is applied to the plankton groups (figure 2, table S4), thus coupling results of variable plankton mortality rates in space and time.

### Simulating climate and fishing scenarios

Among climatic factors, wind is the main driver of the southern Benguela upwelling process. Changes in wind lead to variations in upwelling intensity, thus causing changes in the primary production. According to the optimal environmental window hypothesis [41], we expect the highest phytoplankton productivity when the wind is neither too strong (rapid loss of phytoplankton cells to the open ocean) nor too weak (insufficient input of nutrients from deep water layers). We simulate climate scenarios of increased and decreased wind by using a multiplier (−60%, −30%, no change, +30%, +60%) applied to the monthly values of wind stress at sea surface derived from COADS which force the ROMS model. In this study, only the general trend of wind change is tested, i.e. neither spatial variability nor a higher frequency of extreme wind events has been simulated.

In order to investigate the effects of exploitation on the ecosystem, we designed simple scenarios where changes in fishing pressure only concern the upper part of the food web, i.e. fish that are top predators. In this upwelling ecosystem where small pelagic fish are dominant and targeted, large pelagic and demersal fish also support important fisheries [42], [43], [44] and represent the trophic level classically exploited worldwide. We test variations around the current fishing situation using a multiplier ∈ {0; 1; 2; 3; 4}, applied to the fishing mortality rate of shallow water Cape hake, deep water Cape hake, snoek and silver kob. Small pelagic fish are exploited at their current levels of fishing mortality.

**Figure 3:**
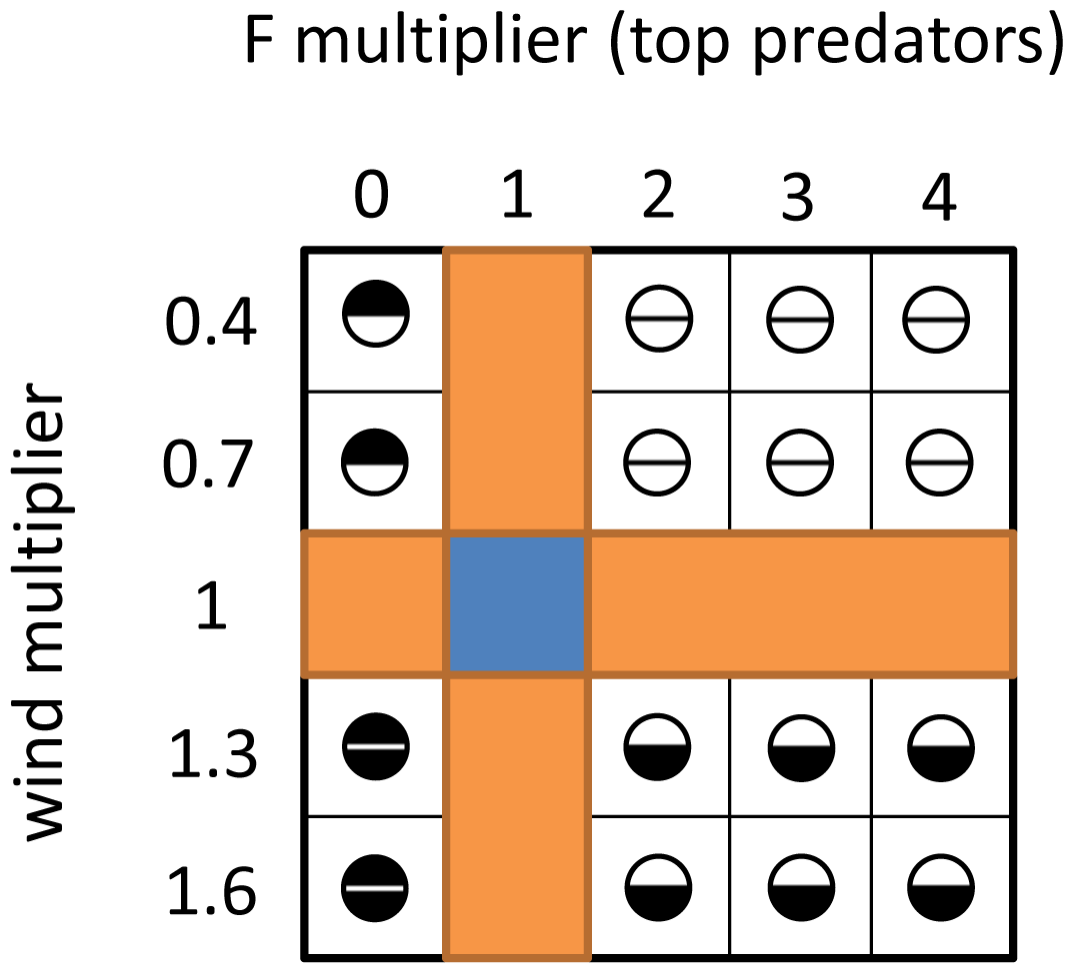
Simulations plan and combination of climate and fishing forcing factors. The blue cell (1;1) corresponds to the current situation of fishing and wind stress forcing. Orange cells correspond to one forcing factor varying and the other factor kept at its current level, i.e. separate effects of fishing (horizontal orange line) and of climate (vertical orange line). The circles represent the simulation of combined effects: the lower half circle represents the bottom-up wind stress forcing, and the upper half circle the top-down fishing pressure. For each half circle, white codes for a negative direct effect (decreased wind stress leads to lower primary production, increased fishing pressure leads to lower biomass of top predator fish), whereas black codes for a direct positive effect.

All 25 combinations of wind and fishing forcing were simulated with ROMS-N_2_P_2_Z_2_D_2_-OSMOSE (figure 3). For each combination of forcing factors, five replicates were run and averaged since OSMOSE is a stochastic model. In this study we do not aim at predicting the ecosystem state under particular wind and fishing pressure, but we explore how fishing and climate effects may combine within the food web. Within this scope we use simple scenarios and track the results at aggregated levels and relative to a baseline simulation. Biomass is computed for four groups: primary producers (dinoflagellates and diatoms), zooplankton (ciliates, copepods and euphausiids), forage fish (anchovy, sardine, redeye, mesopelagic fish and horse mackerel) and top-predator fish (the two species of Cape hake, snoek and silver kob). These four groups are generally used to represent the global trophic levels TL1, TL2, TL3 and TL4 respectively.

From our simulations, we compare the combined effects of fishing and climate (both factors varying simultaneously) with the isolated effects of fishing and climate (one factor varying while the other remains at its current level, figure 3). In order to characterize the type of effect resulting from the combination of fishing and climate, we consider the following definitions:

if the combined effects are equal to the sum of separate effects, they are called additive effects
if the combined effects are greater than the sum of the separate effects, they are considered synergistic or enhanced (either positively or negatively according to the sign of the effect, i.e. an increase or decrease of biomass)
if the combined effects are smaller than the sum of separate effects, they are characterized as dampened.

## Results

**Figure 4:**
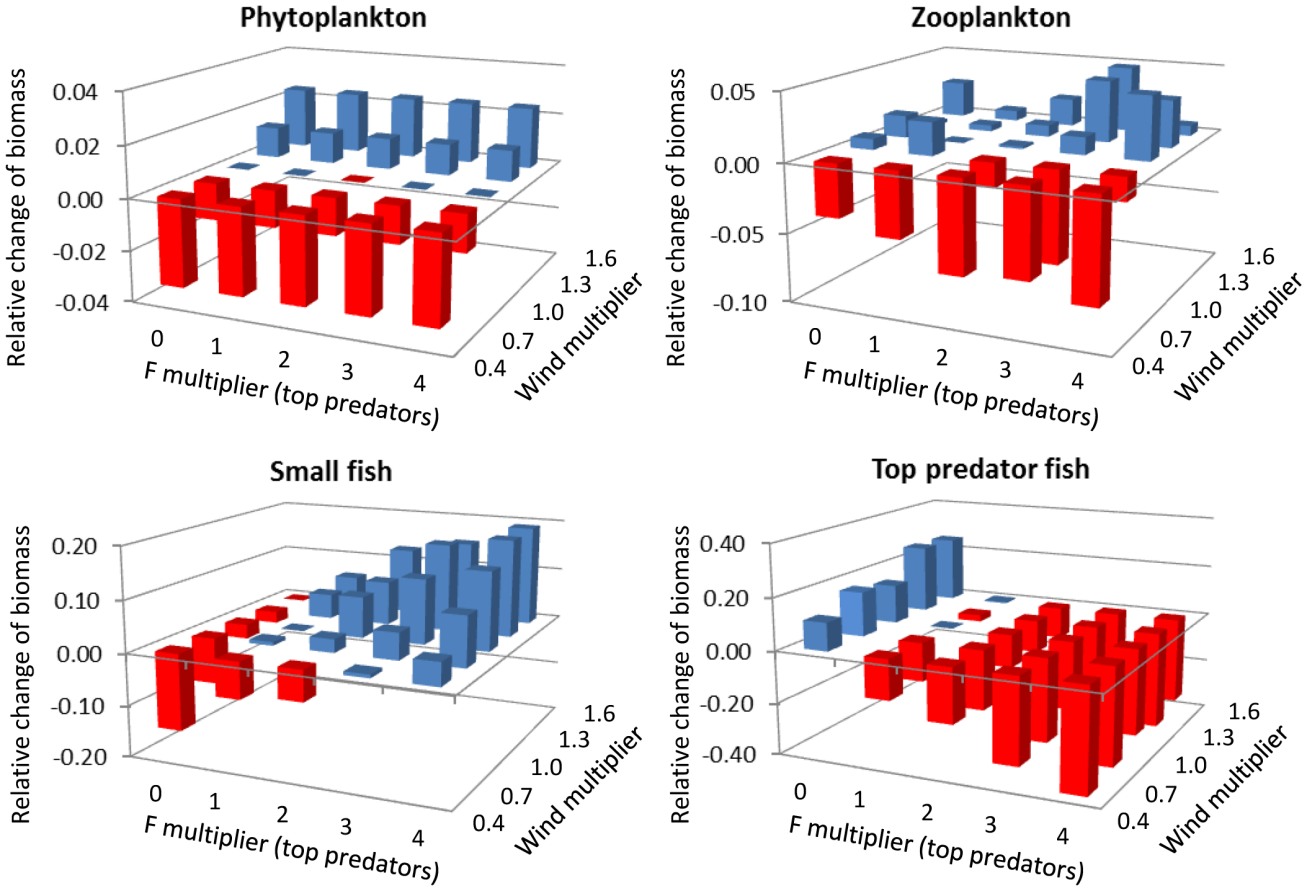
Change of biomass of the four main trophic groups for the 25 sets of simulations. Biomasses are expressed relatively to the baseline situation. Fishing pressure on top predator fish and wind forcing vary according to a multiplier of the baseline values. Blue and red bars represent positive and negative responses, respectively.

For each fishing and wind stress combination, the relative change of biomass of the four trophic groups is calculated (figure 4). The amplitude of variation increases with increasing trophic levels (no more than 4% biomass change for phytoplankton but up to 40% change for top predator fish). Phytoplankton biomass increases with upwelling-favourable wind stress and conversely decreases with decreasing wind stress. Fishing pressure exerted on top predators seems to have no effect on phytoplankton biomass. Zooplankton displays less clear patterns: its biomass is globally higher when the wind stress is stronger but it is not linearly correlated with wind stress intensity as it is for phytoplankton. A strong fishing pressure on top predator fish seems to accentuate wind effects for zooplankton; for low wind stress, there is a stronger biomass decrease at heavy fishing pressure than at low fishing pressure and conversely for high wind stress, there is a stronger biomass increase at heavy fishing pressure than at low fishing pressure. Both an increase in fishing pressure on top predator fish and an increase in upwelling-favourable wind affects the biomass of forage fish positively, while a decrease in both factors leads to a decrease of forage fish biomass. Finally, fishing pressure on top predators leads to a decrease in their biomass. An increase in wind stress does not greatly affect biomass of top predator fish, but leads to an increase of their biomass when they are not heavily fished.

Considered together, the biomass changes of phytoplankton, zooplankton, forage fish and top predators show that the upwelling-favourable wind stress propagates through the food web following bottom-up control. It directly affects phytoplankton, and change in primary production is positively correlated with change in zooplankton biomass, forage fish biomass, and, to some extent, top predator fish biomass (when fishing pressure is low). Conversely, fishing pressure on top predators has top-down effects at a given wind stress that propagate only to forage fish and zooplankton (the trends are opposite between adjacent trophic levels).

**Figure 5:**
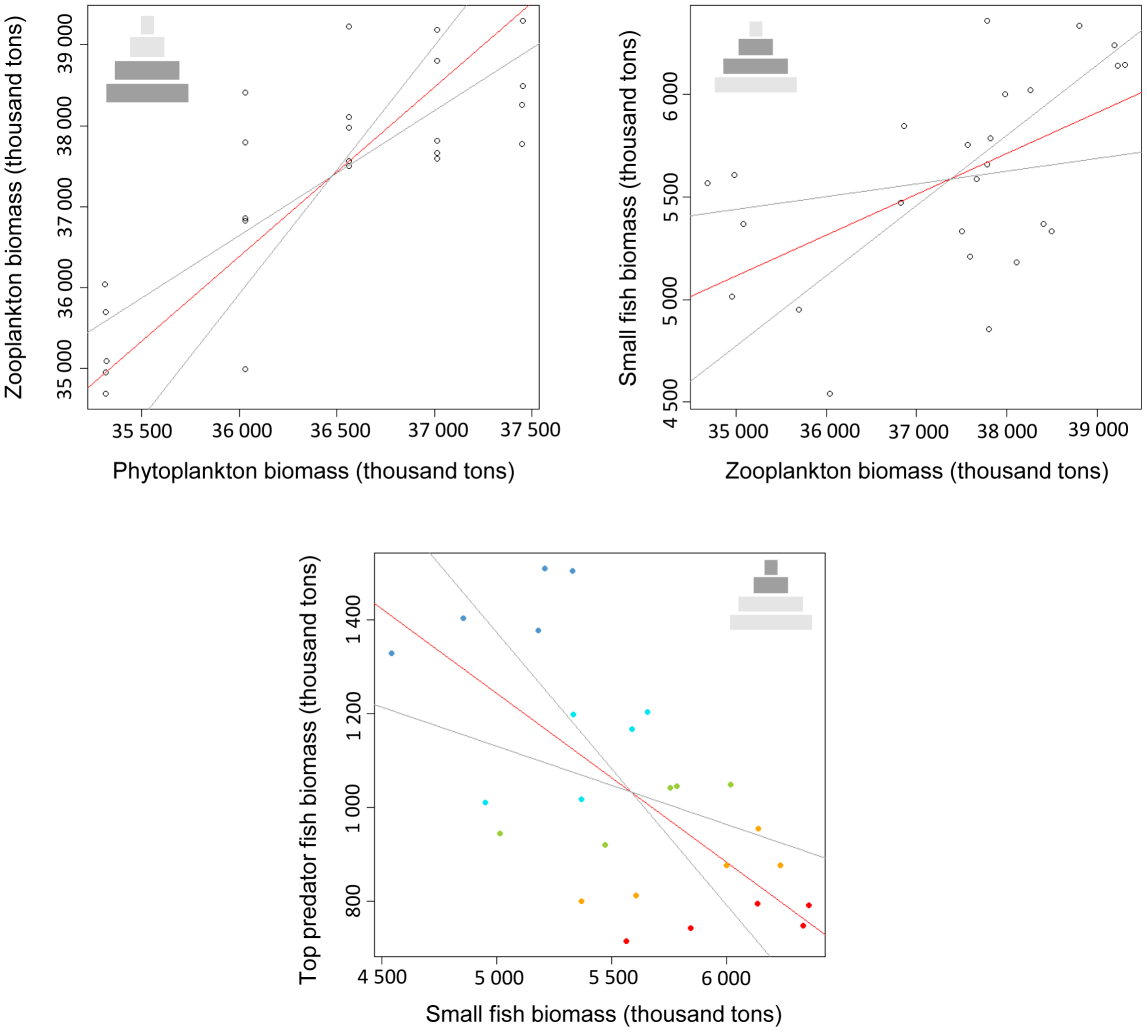
Model II regressions between adjacent trophic levels. Model II regressions are examined between phytoplankton and zooplankton biomass, between zooplankton and small fish biomass, and between small fish and top predator fish biomass. In red, the regression line estimated using major axis regression (MA), and in grey its confidence intervals. On the last graph, coloured dots show the increase of fishing pressure on top predator fish from 0 (dark blue dots) to heavily exploited (red dots). Schematic trophic pyramids are inset top of each plot - model groups regressed are indicated in darker shading.

In order to assess the ecosystem functioning when fishing pressure and upwelling-favourable wind stress act simultaneously, the biomass of each trophic level was compared to the biomass of the next trophic level for each of the 25 simulations (figure 5). Phytoplankton and zooplankton biomasses are correlated positively, illustrating a dominance of bottom-up control between these two groups. The same positive correlation exists between zooplankton biomass and forage fish biomass. Conversely, the biomass of forage fish and the biomass of top predator fish are negatively correlated, indicating the dominance of top-down control between these groups.

**Figure 6:**
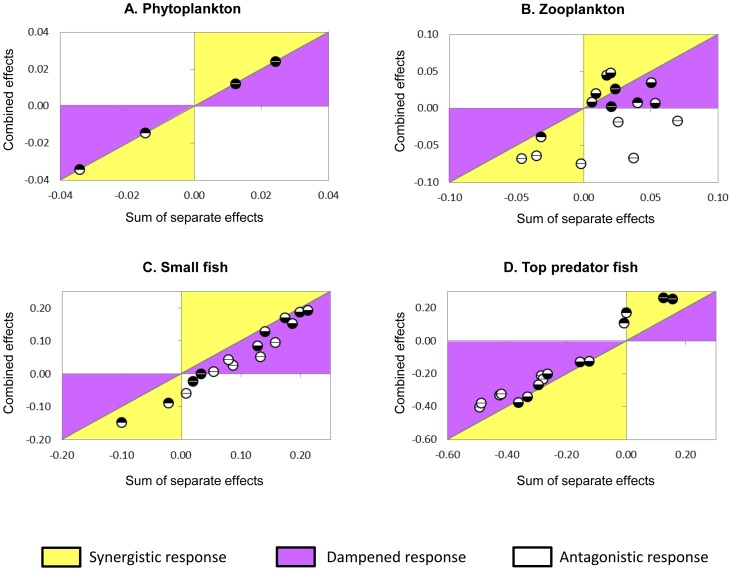
Comparison of combined effects versus separate effects of fishing and climate for each trophic group. Each panel shows relative change of biomass of a trophic group (A: phytoplankton, B: zooplankton, C: small fish, D: top predator fish) when fishing pressure and wind stress act simultaneously (combined effect, y-axis) versus relative change of biomass computed from scenarios of wind stress and fishing pressure acting separately (x-axis). The 1∶1 line represents combined effects equal to the sum of separate effects, i.e. neither synergism nor dampening of effects. The symbols used are the same as in figure 3, each circle corresponding to one of the combined scenarios simulated: the lower half circle represents the bottom-up wind stress forcing, and the upper half circle the top-down fishing pressure. White half-circles code for a negative direct effect (decreased wind stress leads to lower primary production, increased fishing pressure leads to lower biomass of top predator fish), whereas black half-circles represent a direct positive effect. In the yellow area of the plot, the combined effects are amplified compared to the addition of isolated effects; in the purple area, the combined effects are dampened, and in the white area, the combined effects are antagonistic to additional effects.

Finally, the nature of the combined effects of fishing pressure and climate is addressed for each trophic level by comparing the sum of separate effects (hypothesis of additive effects) versus the combined effects resulting from simulations with simultaneous changes in forcing factors (figure 6). As stated above, fishing effects do not propagate down to the phytoplankton level, making this group sensitive only to upwelling-favourable wind. Thus for this functional group, the simulated combined effects are similar to the additive ones, with fishing effects being null (figure 6a).

The three other functional groups react differently to the combined effects of fishing and climate compared to their effects applied separately. Globally, simultaneous changes in fishing pressure on top-predators and wind stress lead to a reduced biomass of zooplankton compared to predictions from separate effects. However, the type of combined effects at the zooplankton level is ambiguous: for 2 simulations they are additive (1∶1 line, figure 6b), for 7 simulations they are synergistic (3 positively, i.e. larger biomass increase than expected, and 4 negatively, i.e. larger biomass decrease than expected), they are dampened for 4 simulations (smaller biomass increase than expected) and they are antagonistic to additional effects for 3 simulations. The pattern is clearer for small pelagic fish, where all combined scenarios result in lower biomass than expected from the isolated drivers (figure 6c). Most of the combined simulations illustrate dampened effects of fishing and climate acting simultaneously. However, when both wind stress and fishing pressure on top predator fish are low, the decrease in small pelagic fish biomass is stronger than simulated from separate effects, which indicates a synergistic reaction. Conversely, at the level of top predator fish, all combined simulations result in higher biomass of top predators than expected from separate effects (figure 6d). Mostly, they correspond to dampened negative effects of fishing pressure and wind forcing compared to the additive effects. When fishing pressure on top predators decreases, there is also positive synergistic effects as the biomass increase of top predator fish is higher than predicted from separate drivers.

## Discussion

Regarding trophic controls that may operate in the southern Benguela ecosystem, the simulations produced expected results; simulating an increase in upwelling-favourable wind stress leads to an increased biomass of phytoplankton, zooplankton, small pelagic fish, as well as top predator fish when they are under no or moderate exploitation. This illustrates the bottom-up effect of climate propagating up the food chain [45]. On the other hand, an increased fishing pressure on top predators leads to a decrease of their biomass and an increase of small pelagic fish biomass. This effect cascades down to zooplankton, which decreases in biomass at low wind stress, but dampens before it reaches the phytoplankton level, at which biomass does not change. The intensity of trophic controls may depend on the level of primary production of the system [46], the abundance of top predators [47] and their diversity [46]. The high primary production of the Benguela upwelling could explain the low propagation of top-down effects of fishing pressure. In our simulations, there are limited top-down effects of fishing propagating down to zooplankton, occurring only when wind stress and thus primary production are reduced. This propagation of fishing effects down to low trophic levels, which is surprising in such a productive system, can be explained by the non-selective fishing scenario applied, i.e. fishing pressure affects all top predator fish, which prevents the dampening of top-down control by the diversity of top predators [48].

To characterize the combined effects of fishing and climate, we deliberately chose to simulate relatively simple scenarios which were mainly used to determine the demographic effects of fishing and climate. However, as fishing directly impacts recruited (i.e. larger) individuals, the size structure of the fish community is also altered. In parallel, the bottom-up effects of wind stress on the primary production will also affect predation up the food web, and hence the growth rate and size of fish. Because pelagic ecosystems are highly size-structured, fishing-and climate-induced changes in body size impact trophic interactions and thus food web dynamics [49]. Whereas those are explicitly accounted for in OSMOSE, other additional effects on life-history traits and their propagation through the food web could be considered. For instance, evolutionary effects of fishing could be added through varying fish condition and maturation parameters [50], [51]. Climate effects could be broadened to include, among others [52], the temperature effects on physiological rates but also evolutionary change in spawning date [53] or migration patterns [54]. End-to-end models can inform us about the relative contribution of each effect on ecosystem functioning, by switching them on and off, and as an extension of this study they can also be useful for investigating how all these effects are combined through food web dynamics.

Using an end-to-end model as a virtual laboratory informs us about the dominant trophic control structuring the food web. In the coupled model ROMS-N_2_P_2_Z_2_D_2_-OSMOSE, predation is completely opportunistic, depending only on spatio-temporal co-occurrence and size suitability between prey and predator. Thus the dominance of trophic controls emerges from local individual interactions, and is not set *a priori*. Our results will be helpful for alternative modelling studies, where trophic controls influence simulation results (e.g. [55]) and must be set and parameterized carefully. The comparison of biomass changes between adjacent trophic levels under several intensities of wind forcing and fishing pressure allows us to assess the dominance of bottom-up versus top-down controls. In the scenarios examined here, the lower part of the food chain from phytoplankton to forage fish is predominantly driven by bottom-up control by upwelling-favourable winds. Top-down control dominates the relationship between top-predators and forage fish, the latter becoming the “meeting point” of bottom-up and top-down controls. Forage species are considered a key functional group in upwelling systems that are usually driven by wasp-waist control, i.e. bottom-up effect of climate from forage fish to top predators and top-down effect of forage fish on zooplankton [56]. How the effects of fishing and climate propagate through a food web will depend to a large extent on which trophic level the climate and fishing forcing are specifically acting (e.g. by comparison, Shannon et al. [29] considered both climate and fishing acting at the forage fish trophic level). Further, the low diversity of this highly abundant functional group has been discussed [57] and, irrespective of whether wasp waist flow controls or converging flow controls operate, this functional group appears to play a key role in structuring the food web of the Benguela ecosystem.

As stated by Perry et al. [58], “modern fisheries research and management must understand and take account of the interactions between climate and fishing, rather than try to disentangle their effects and address each separately”. Several studies have recently looked at the combination of multiple stressors on aquatic ecosystems [59], [60], [61], [16], [17], [18] in order to describe them as additive (or multiplicative), synergistic (also called amplified) or antagonistic (reduced, dampened). However, no consensus has been reached concerning the nature of the combined effects of fishing pressure and climate, because it may depend on the ecosystem considered, the climatic drivers tested, the trophic level directly impacted by the climatic or fishing driver, the indicators analysed, etc… Here we show that fishing pressure and upwelling-favourable wind stress have mostly dampened combined effects on fish populations. However, these dampened effects are expressed differently between small pelagic fish and top predator fish. The latter benefit from all combinations of fishing and climate pressure, as illustrated by their higher biomass simulated when both stressors act simultaneously. Conversely to other studies where heavy fishing pressure is considered to render fish populations more sensitive to climate [62], our simulations suggest that there is no synergistic negative effects on top predator fish when both fishing and climate act together. In contrast, the dampened effects observed at the forage fish level result in lower biomass than expected under isolated drivers, suggesting that this functional group should be managed carefully, as diagnoses considering fishing pressure only might be more optimistic than under combined effects with the environmental variability. It is important to remember that the southern Benguela upwelling system is unusual in that it shows a relatively low biomass of small pelagic fish compared to other upwelling ecosystems, and relative to its high level of primary production [63]. Our results suggest that their relatively low biomass is linked to the combination of fishing and climate forcing. It might also be related to the fact that forage fish constitute the meeting point of top-down and bottom-up controls (converging controls). This study emphasizes the need for close monitoring of small pelagic fish, which appear to be structurally important in the trophic functioning and energy flows in the Benguela upwelling ecosystem and very sensitive to climate and fishing pressures.

## Supporting Information

Table S1 **Equations representing the main processes in the N_2_P_2_Z_2_D_2_ model.** *[P]* represents the concentration of phytoplankton in mmol.N.m^−3^, *[Z]* represents the concentration of zooplankton in mmol.N.m^−3^, *[D]* is set for detritus and *[NH_4_]* and *[NO_3_]* are for ammonium and nitrate. Parameter values used for each group can be found in Koné *et al.*, 2005.
(DOC)

Table S2 **Equations representing the main processes in the OSMOSE model.** *N_i,t_* and *B_i,t_* are respectively the abundance and the biomass of a school *i* at time *t*.
(DOC)

Table S3 **Input parameters of the OSMOSE model for the 11 fish species modelled explicitly.** *L_∞_*, *K*, and *t_0_* are the parameters of the von Bertalanffy growth model; *c* is Fulton's condition factor and *b* the exponent of the L-W allometric relationship; *φ* is relative fecundity; *a_mat_* is age at maturity; *a_max_* is longevity; *M_add_* is an additional mortality rate (resulting from predation by other species of the ecosystem that are not explicitly modelled); *F* is the annual fishing mortality rate; *a_rec_* is age of recruitment; *L_thr_* is the size threshold separating two sets of predation ratios, for the larvae and juveniles organisms (Lar/Juv) and for adults.
(DOC)

Table S4 **Formulation of the predation process used for coupling models, using plankton as food for the predation in OSMOSE, and applying a predation mortality rate field in ROMS-N_2_P_2_Z_2_D_2_ according to the plankton biomass effectively eaten.** *BE_i,p,Δt_* is the biomass of plankton group *p* eaten by the school *i* during the time step *Δt*, *m_HTL_* is the HTL-induced mortality rate. See Travers-Trolet *et al.* (in press) for more details.
(DOC)
