# Supplementary material for: Combined Fishing and Climate Forcing in the Southern Benguela Upwelling Ecosystem: An End-to-End Modelling Approach Reveals Dampened Effects
Source: PLoS One. 2014 Apr 7;9(4):e94286. doi: 10.1371/journal.pone.0094286 (PMC3978043; doi:10.1371/journal.pone.0094286)
Supplement: Table S1 — Equations representing the main processes in the N2P2Z2D2 model. [P] represents the concentration of phytoplankton in mmol.N.m−3, [Z] represents the concentration of zooplankton in mmol.N.m−3, [D] is set for detritus and [NH4] and [NO3] are for ammonium and nitrate. Parameter values used for each group can be found in Koné et al., 2005. (DOC) [file pone.0094286.s001.doc]

**Table S1: Equations representing the main processes in the N_2_P_2_Z_2_D_2_ model.** *[P]* represents the concentration of phytoplankton in mmol.N.m^-3^, *[Z]* represents the concentration of zooplankton in mmol.N.m^-3^, *[D]* is set for detritus and *[NH_4_]* and *[NO_3_]* are for ammonium and nitrate. Parameter values used for each group can be found in [Ko*né et a*l., 2005](#_ENREF_1).

| **Processes** | **Equations** | **Parameters** |
| --- | --- | --- |
| **Nutrient uptake** | $\mu_{p}(N)=\frac{[NH_{4}]}{k_{NH_{4}}+[NH_{4}]}+\left(\frac{[NO_{3}]}{k_{NO_{3}}+[NO_{3}]}.\frac{k_{NH_{4}}}{k_{NH_{4}}+[NH_{4}]}\right)$ | k_NH4_ and K_NO3_ are the half-saturation constants |
| **Phytoplankton growth** | $\mu_{p}=\mu_{p}(PAR,T)^{\mathop{}\limits}.\mu_{p}(N)$ | μ_p_(PAR, T) is the light and temperature limitation factor |
| **Predation** | $g_{z}=g_{\text{max}}.\frac{\sum e_{zi}.[F_{i}]}{k_{z}+\sum e_{zi}.[F_{i}]}$ | *g_max_* is the maximum grazing rate, *e_zi_*  are preference coefficients for different prey *i, k_z_* is the half-saturation constant, and [*F_i_*] is the concentration of prey *i*. |
| **Egestion** | $Eg=(1-\beta).g_{z}.[Z]$ | *β* is the assimilation coefficient |
| **Excretion** | $Ex_{NH_{4}}=\mu_{Z}.[Z]$ | Excretion rate (*µ_Z_*) |
| **Natural mortality** | $M_{P}=-m_{P}.[P]$ $M_{Z}=-m_{Z}.[Z]$ | Mortality rates (*m_P_* and *m_Z_*) |
| **Remineralisation** | $\mathrm{Re}_{NH_{4}}=\mu_{D}.[D]$ | Remineralization rate (*µ_D_*) |
| **Nitrification** | $Ni_{NO_{3}}=\mu_{AN}[NH_{4}]$ | Nitrification rate (*µ_AN_*) |
