# Supplementary material for: Combined Fishing and Climate Forcing in the Southern Benguela Upwelling Ecosystem: An End-to-End Modelling Approach Reveals Dampened Effects
Source: PLoS One. 2014 Apr 7;9(4):e94286. doi: 10.1371/journal.pone.0094286 (PMC3978043; doi:10.1371/journal.pone.0094286)
Supplement: Table S2 — Equations representing the main processes in the OSMOSE model. Ni,t and Bi,t are respectively the abundance and the biomass of a school i at time t. (DOC) [file pone.0094286.s002.doc]

**Table S2: Equations representing the main processes in the OSMOSE model.** *N_i,t_* and *B_i,t_* are respectively the abundance and the biomass of a school *i* at time *t*.

| **Processes** | **Equations** |
| --- | --- |
| **Predation** | Successively for each school *i*, in each cell, and for prey *j* of suitable size, $PB_{i,j,\Delta t}=a_{j}.B_{j,t}*\min\left(1,\frac{r*B_{i,t}}{\sum a_{j}.B_{j,t}}\right)$ With *PB_i,j,Δt_* the biomass of the school *j* preyed upon by the school *i*, *a_j_* the availability coefficient of the prey *j* and *r* the maximum predation rate, set for all species to 3.5g of prey per gram of body mass per year |
| **Growth** | $\left\{\begin{matrix} \Delta L_{i,t}=0 & if\xi_{i}<\xi_{crit} \\ \Delta L_{i,t}=\frac{2\Delta L}{1-\xi_{crit}}\left(\xi_{i}-\xi_{crit}\right) & if\xi_{i}>\xi_{crit} \end{matrix}\right.$ *ΔL* is the mean growth rate calculated from the von Bertalanffy model at the appropriate length and *ΔL_i,t_* is the actual growth rate of the school *i* at the time step *t*. *ξ_i._* is the predation efficiency and corresponds to (*PB_i,j,Δt_/r B_i,t_*) and *ξ_crit_* its critical value set for all species at 0.57 |
| **Starvation mortality** | $M_{\xi}=\frac{-M_{\xi\max}}{\xi_{crit}}.\xi_{i}+M_{\xi\max}$ $N_{S,i}=N_{i,t}(1-e^{-\Delta t.M_{\xi}})$ *N_S,i_* is the number of fish from the school *i* which starve and die. *M_ξmax_* is the maximum starvation mortality rate set for all species at 1 year^-1^ |
| **Fishing mortality** | $N_{i,t+\Delta t}=N_{i,t}.e^{-\Delta t.F(t)}$ with F the fishing mortality rate |
| **Additional mortality** | $N_{i,t+\Delta t}=N_{i,t}.e^{-\Delta t.M}$ with M the additional mortality rate |
| **Reproduction** | $N_{0}=\Phi.\frac{1}{2}\sum_{a>A_{mat}} B_{a,t}$ *N_0_* is the number of eggs released in the system, *Ф* is the relative fecundity  and *A_mat_* is the age at maturity |
