# Supplementary material for: Combined Fishing and Climate Forcing in the Southern Benguela Upwelling Ecosystem: An End-to-End Modelling Approach Reveals Dampened Effects
Source: PLoS One. 2014 Apr 7;9(4):e94286. doi: 10.1371/journal.pone.0094286 (PMC3978043; doi:10.1371/journal.pone.0094286)
Supplement: Table S3 — Input parameters of the OSMOSE model for the 11 fish species modelled explicitly. L∞, K, and t0 are the parameters of the von Bertalanffy growth model; c is Fulton's condition factor and b the exponent of the L-W allometric relationship; φ is relative fecundity; amat is age at maturity; amax is longevity; Madd is an additional mortality rate (resulting from predation by other species of the ecosystem that are not explicitly modelled); F is the annual fishing mortality rate; arec is age of recruitment; Lthr is the size threshold separating two sets of predation ratios, for the larvae and juveniles organisms (Lar/Juv) and for adults. (DOC) [file pone.0094286.s003.doc]

**Table S3: Input parameters of the OSMOSE model for the 11 fish species modelled explicitly. *L*_∞_, *K*, and *t*_0_ are the parameters of the von Bertalanffy growth model; *c* is Fulton’s condition factor and *b* the exponent of the L-W allometric relationship; *φ* is relative fecundity; *a*_mat_ is age at maturity; *a*_max_ is longevity; *M_add_* is an additional mortality rate (resulting from predation by other species of the ecosystem that are not explicitly modelled); *F* is the annual fishing mortality rate; *a*_rec_ is age of recruitment; *L_thr_* is the size threshold separating two sets of predation ratios, for the larvae and juveniles organisms (Lar/Juv) and for adults.**

|  | **Growth** | | | | | **Reproduction** | | **Survival** | | | | **Predation** | | | | | |
| --- | --- | --- | --- | --- | --- | --- | --- | --- | --- | --- | --- | --- | --- | --- | --- | --- | --- |
|  | *L_∞_* | *K* | *t_0_* | *c* | *b* | *φ* | *a_mat_* | *a_max_* | *M_add_* | *F* | *a_rec_* | *L_thr_* | Min ratio | | | Max ratio | |
| Species | cm | y^-1^ | y | g.cm^-3^ |  | eggs.g^-1^ | y | y | y^-1^ | y^-1^ | y | cm | Larv/Juv | | Adult | Larv/Juv | Adult |
| Euphausiids | 1.84 | 1.682 | -0.1975 | 0.00738 | 3.16 | 42254 | 0.3 | 1 | 0.1 | 0 | - | 0.6 | 5 | | 5 | 1000 | 500 |
| Anchovy | 14.8 | 1.37 | -0.03 | 0.007 | 3 | 8000 | 1 | 5 | 0.403 | 0.23 | 1 | 8 | 3.5 | | 3.5 | 100 | 500 |
| Sardine | 26 | 0.26 | -1.5 | 0.009 | 3 | 2400 | 2 | 10 | 0.365 | 0.16 | 1 | 10 | 4 | | 100 | 100 | 10000 |
| Redeye | 30.1 | 0.71 | 0.28 | 0.009 | 3 | 750 | 1 | 6 | 0.208 | 0.04 | 1 | **-** | 4 | | | 80 | |
| Lanternfish | 7 | 1.66 | 0.06 | 0.008 | 3 | 646 | 0.5 | 2 | 0.226 | 0.0003 | 1 | **-** | 3.5 | | | 80 | |
| Lightfish | 6 | 1.15 | 0.06 | 0.008 | 3 | 334 | 0.5 | 2 | 0.226 | 0.0003 | 1 | **-** | 3.5 | | | 80 | |
| Horse mackerel | 54.5 | 0.183 | -0.65 | 0.009 | 3 | 250 | 3 | 8 | 0.314 | 0.06 | 2 | **-** | 5 | | | 100 | |
| Shallow water hake | 270.6 | 0.039 | -0.73 | 0.006543 | 3.0425 | 500 | 4 | 15 | 0.228 | 0.23 | 3 | 27 | 3 | 1.8 | | 30 | 30 |
| Deep water hake | 219.4 | 0.049 | -0.914 | 0.007846 | 2.9759 | 500 | 4 | 15 | 0.174 | 0.33 | 3 | 29 | 3 | 1.8 | | 30 | 30 |
| Snoek | 115.3 | 0.294 | -0.1 | 0.018 | 3 | 130 | 3 | 10 | 0.132 | 0.25 | 2 | **-** | 3 | | | 30 | |
| Silver kob | 116 | 0.12 | -1.47 | 0.007 | 3 | 150 | 2 | 25 | 0.228 | 0.181 | 3 | **-** | 3 | | | 30 | |
