# Supplementary material for: Combined Fishing and Climate Forcing in the Southern Benguela Upwelling Ecosystem: An End-to-End Modelling Approach Reveals Dampened Effects
Source: PLoS One. 2014 Apr 7;9(4):e94286. doi: 10.1371/journal.pone.0094286 (PMC3978043; doi:10.1371/journal.pone.0094286)
Supplement: Table S4 — Formulation of the predation process used for coupling models, using plankton as food for the predation in OSMOSE, and applying a predation mortality rate field in ROMS-N2P2Z2D2 according to the plankton biomass effectively eaten. BEi,p,Δt is the biomass of plankton group p eaten by the school i during the time step Δt, mHTL is the HTL-induced mortality rate. See Travers-Trolet et al. (in press) for more details. (DOC) [file pone.0094286.s004.doc]

**Table S4: Formulation of the predation process used for coupling models, using plankton as food for the predation in OSMOSE, and applying a predation mortality rate field in ROMS-N_2_P_2_Z_2_D_2_ according to the plankton biomass effectively eaten.** *BE_i,p,Δt_* is the biomass of plankton group *p* eaten by the school *i* during the time step *Δt, m_HTL_* is the HTL-induced mortality rate. See Travers-Trolet et al. (in press) for more details.

| **Processes** | **Equations** |
| --- | --- |
| **Predation** | Successively for each school *i*, in each cell *(x,y)* $BE_{i,p,\Delta t}(x,y)=a_{p}.B_{p,t}.sr_{i,p}.\frac{TBE_{i,\Delta t}}{TBA_{i,\Delta t}}$ With *a_p_* the accessibility coefficient of the plankton group *p*, s*r_i,p_* the percentage of the plankton size range available to the school *i* according to size suitability, *TBE_i,Δt_* the total biomass of prey (both plankton and fish) eaten by the school *i* during the time step *Δt* and *TBA_i,Δt_* the total biomass of prey (both plankton and fish) available to the school *i* during the time step *Δt* |
| **Predation mortality** | In each cell (*x,y*) and for each plankton group (*p*) $m_{HTL}(x,y,t+\Delta t,p)=\frac{\sum BE_{i,p,\Delta t}(x,y)}{\Delta t.B_{p,t}(x,y)}$ With *B_p,t_ (x,y)* the accessible plankton biomass |
